# Metformin Inhibits Na^+^/H^+^ Exchanger NHE3 Resulting in Intestinal Water Loss

**DOI:** 10.3389/fphys.2022.867244

**Published:** 2022-04-04

**Authors:** Yiran Han, C. Chris Yun

**Affiliations:** ^1^ Gastroenterology Research, Atlanta Veterans Administration Medical Center, Decatur, GA, United States; ^2^ Division of Digestive Diseases, Department of Medicine, Emory University School of Medicine, Atlanta, GA, United States; ^3^ Winship Cancer Institute, Emory University School of Medicine, Atlanta, GA, United States

**Keywords:** AMPK, intestinal epithelial cell, Nedd4-2, NHE3, type 2 diabetes, ubiquitin

## Abstract

Glycemic control is the key to the management of type 2 diabetes. Metformin is an effective, widely used drug for controlling plasma glucose levels in diabetes, but it is often the culprit of gastrointestinal adverse effects such as abdominal pain, nausea, indigestion, vomiting, and diarrhea. Diarrhea is a complex disease and altered intestinal transport of electrolytes and fluid is a common cause of diarrhea. Na^+^/H^+^ exchanger 3 (NHE3, *SLC9A3*) is the major Na^+^ absorptive mechanism in the intestine and our previous study has demonstrated that decreased NHE3 contributes to diarrhea associated with type 1 diabetes. The goal of this study is to investigate whether metformin regulates NHE3 and inhibition of NHE3 contributes to metformin-induced diarrhea. We first determined whether metformin alters intestinal water loss, the hallmark of diarrhea, in type 2 diabetic *db/db* mice. We found that metformin decreased intestinal water absorption mediated by NHE3. Metformin increased fecal water content although mice did not develop watery diarrhea. To determine the mechanism of metformin-mediated regulation of NHE3, we used intestinal epithelial cells. Metformin inhibited NHE3 activity and the effect of metformin on NHE3 was mimicked by a 5′-AMP-activated protein kinase (AMPK) activator and blocked by pharmacological inhibition of AMPK. Metformin increased phosphorylation and ubiquitination of NHE3, resulting in retrieval of NHE3 from the plasma membrane. Previous studies have demonstrated the role of neural precursor cell expressed, developmentally down-regulated 4-2 (Nedd4-2) in regulation of human NHE3. Silencing of Nedd4-2 mitigated NHE3 inhibition and ubiquitination by metformin. Our findings suggest that metformin-induced diarrhea in type 2 diabetes is in part caused by reduced Na^+^ and water absorption that is associated with NHE3 inhibition, probably by AMPK.

## Introduction

Diabetes mellitus, a common consequence of the growing obesity epidemic, has emerged as a global health threat. Gastrointestinal (GI) adverse effects such as abdominal pain, nausea, dyspepsia, anorexia, and diarrhea are common side effects affecting up to 76% of patients with diabetes (Feldman and Schiller, 1983; Krishnasamy and Abell, 2018). Diarrhea is more common in young to middle-aged patients with poorly controlled insulin-requiring type 1 diabetes (T1D) (Ogbonnaya and Arem, 1990). In type 2 diabetes (T2D), diarrhea is less frequent, but medications are often a culprit for chronic diarrhea (Dandona et al., 1983; Foss and Clement, 2001; Gould and Sellin, 2009). Metformin is a highly effective drug to treat T2D, but the high incidence of adverse effects, including diarrhea, limits mediation adherence amongst patients with T2D (Dandona et al., 1983; Foss and Clement, 2001; McGovern et al., 2018). The mechanism of diarrhea resulting from taking metformin are not clear, but several explanations, including stimulation of secretion serotonin and histamine, malabsorption of bile acids, and bacterial overgrowth have been suggested (McCreight et al., 2016).

Metformin is the most widely prescribed drug for treatment of T2D. Its glucose lowering effect results from decreased hepatic glucose production and increased glucose utilization (Davidson and Peters, 1997). Metformin indirectly activates 5′-AMP-activated protein kinase (AMPK) by inhibiting mitochondrial complex I of the electron transport chain to increase the intracellular AMP/ATP ratio (Owen et al., 2000; Zhou et al., 2001). AMPK is a cellular and whole-body energy sensor involved in the regulation of energy homeostasis (Luo et al., 2005). Dysregulation of AMPK has been observed in metabolic diseases including T2D (Coughlan et al., 2014). Skeletal muscle contraction and myocardial ischemia activate AMPK and the benefit of exercise in diabetic patients is well known (Musi et al., 2001). AMPK is expressed in the intestinal epithelial cells (IECs) where deletion of AMPK alters epithelial barrier function (Harmel et al., 2014; Sun et al., 2017; Olivier et al., 2021). AMPK is a heterotrimer, consisting of *α* catalytic subunit, and *ß* and *γ* regulatory subunits. AMPK is activated by phosphorylation at T172 of the *α* subunit by upstream kinases, such as liver kinase B1 and calmodulin-dependent protein kinase kinase-2. Once active, AMPK switches off energy-consuming pathways, such as fatty acid synthesis, by phosphorylating and inhibiting acetyl CoA carboxylase, and protein translation via inhibition of the mammalian target of rapamycin pathway (Xiao et al., 2011). Metformin can increase glucose uptake and expression levels of sodium-coupled glucose cotransporter 1 (SGLT1) in rat intestine (Bailey et al., 1994; Lenzen et al., 1996).

Previous studies have shown that AMPK inhibits ion transport. Activation of AMPK by metformin inhibits the epithelial Na channel (ENaC) and the cystic fibrosis transmembrane regulator (CFTR) in epithelial cells derived from the lung, which may benefit by reducing excessive sodium loading and inflammation associated with CF (Hallows et al., 2003; Carattino et al., 2005; Myerburg et al., 2010). Metformin increases urine osmolality in a rat model of congenital diabetes insipidus by stimulating urea transporter (UT-A1), aquaporin 2 (AQP2), and Na^+^-K^+^-2Cl^-^ cotransporter 2 (NKCC2) (Efe et al., 2016; Klein et al., 2016). In the intestine, AMPK inhibits Cl^−^ secretion through inhibition of CFTR and Na^+^-K^+^-2Cl^-^ cotransporter 1 (NKCC1) (Rogers et al., 2013; King et al., 2019).

Diarrhea often results from malabsorption of injected food or an imbalance between electrolyte absorption and secretion (Surawicz, 2010). Na^+^/H^+^ exchanger 3 (NHE3, SLC9A3), expressed in the apical membrane of the small intestine and proximal colon (Hoogerwerf et al., 1996), mediates the majority of Na^+^ absorption between meals and is a frequent target of enteropathogens leading to diarrhea. Mice lacking NHE3, either globally or intestine-specific, develop chronic diarrhea (Schultheis et al., 1998; Xue et al., 2020). Reduced NHE3 expression has been reported in a mouse model of colitis and patients with inflammatory bowel disease (Barmeyer et al., 2004; Siddique et al., 2009; Sullivan et al., 2009; Janecke et al., 2016). Patients with mutations in the *Slc9A3* gene that reduce NHE3 expression develop congenital diarrhea (Janecke et al., 2015). Our recent study of T1D has shown that NHE3 expression is downregulated in T1D humans and mice, and insulin stimulates NHE3 activity by restoring NHE3 expression in the brush border membrane of T1D mouse intestine (He et al., 2015). In the current study, we hypothesized that AMPK inhibits NHE3 and contributes to diarrhea in T2D associated with metformin treatment. We used *db/db* T2D mice to demonstrate intestinal water loss by metformin. Our data show that AMPK inhibits NHE3 activity, and this regulation is dependent on ubiquitination by the E3 ubiquitin ligase, neural precursor cell-expressed, developmentally down-regulated 4–2 (Nedd4-2).

## Materials and Methods

### Reagents and Antibodies

Metformin, 5-aminoimidazole-4-carboxamide ribonucleoside (AICAR), dorsomorphin (Compound C) were purchased from Sigma-Aldrich (St. Louis, MO). Rabbit polyclonal anti-NHE3 antibody EM450 and mouse monoclonal anti-VSVG P5D4 were previously described (Yoo et al., 2012). The following antibodies were commercially obtained: anti-NHE3 (NHE31-A, Alpha Diagnostics, San Antonio, TX)), Anti-phospho-NHE3 (#MABN2415; MilliporeSigma, Burlington, MA)), anti-HA (C29F4, Cell Signaling, Danvers, MA), anti-phospho-S342-Nedd4-2 (12,146, Cell Signaling), anti-phospho-S448-Nedd4-2 (8,063, Cell Signaling), anti-ubiquitin (P5D1, Santa Cruz, Dalla, TX), anti-Nedd4L (46,521, Abcam, Waltham, MA), and anti-*β*-actin (4,967, Cell Signaling) antibodies.

### Cell Culture and Plasmids

Caco-2bbe and SK-CO15 cells were cultured as previously described (Yoo et al., 2012). pcDNA3.1 construct carrying human NHE3 (pcDNA-hNHE3V) with a C-terminal vesicular stomatitis virus glycoprotein (VSVG) epitope has been described (No et al., 2014). Caco-2bbe cells transfected with pcDNA-hNHE3V, Caco-2bbe/NHE3, were previously generated (Lin et al., 2010). The lentiviral plasmid pLKO.1 containing short hairpin RNA (shRNA) targeting Nedd4-2 (shNedd4-2) or a scrambled control shRNA (shCon) was purchased from Sigma.

### Animals and Metformin Treatments

Five-six weeks old male *Lepr* ^*db/db*^ (*db/db*) mice were obtained from the Jackson laboratory (Bar Harbor, ME). Mice were acclimated and given standard chow and water ad libitum for 2 weeks before the start of experiments. Mice were divided into two groups: the experimental group receiving metformin in their drinking water (1.0 mg/ml) for 30 days, and the control group receiving normal tap water. On day 25, 27 and 29, metformin at the concentration of 500 mg/kg was administered once a day by gavage using a 4 cm-long curved needle with a steel ball at the tip. Blood samples were collected, and glucose levels were measured with a OneTouch Verio glucose meter (LifeScan, Malvern, PA)) every 4–5 days. At the same time, body weights were measured. To determine fecal water contents, feces were collected for 1 h at the end of the experimental period, and total fecal weight was measured. Fecal weight was measured again after overnight dehydration, and fecal water content was determined by (fecal weight before dehydration - fecal weight after dehydration)/fecal weight before dehydration X 100.

### Intestinal Water Flux Measurement

Intestinal water flux was measured as previously reported (Lin et al., 2010). Briefly, a mouse anaesthetized with sodium pentobarbital was placed on a 37°C heating block, and a small incision was made in the abdomen. A 5-cm loop of the ileum (between 5 and 10 cm upstream of cecum) cannulated at the proximal and distal ends was flushed with saline for 10 min. This was followed by perfusion of pre-warmed perfusion solution (mM: 118.4 NaCl, 4.7 KCl, 2.52 CaCl_2_, 1.18 MgSO_4_, 25 Na gluconate, 1.18 KH_2_PO_4_, pH 7.4) at 1 ml/min for 2 h. The CFTR inhibitor CFTRinh-172 (250 ug/kg) was administered by intraperitoneal injection to each mouse 1 h prior to the start of perfusion. Intestinal water flux was recorded by calculating a change in the volume of perfusion buffer in a reservoir every 10 min over the course of a 90–120 min perfusion period.

### Na^+^ Dependent Intracellular pH Recovery

The Na^+^-dependent changes in intracellular pH (pH_i_) by NHE3 was determined using the ratio-fluorometric, pH-sensitive dye 2′,7′-bis-(2-carboxyethyl)-5-carboxyfluorescein acetoxymethyl ester (BCECF-AM) as previously described (Yoo et al., 2012). Caco-2bbe cells grown for 10–14 days post-confluence were treated with 1 mM metformin or the AMPK activator AICAR (500 μM) 30 min during dye loading. For the measurement of NHE3 activity in mouse intestine, isolation of villi from mice ileum was performed as previously described (He et al., 2011). In brief, mice were euthanized with isoflurane and the ileum was flushed with cold PBS to remove food particles. An equivalent segment of the proximal ileum (approximately 10 cm upstream of the cecum) was opened longitudinally and stabilized on a cooled stage. The villi were dissected under stereomicroscope using sharpened microdissection scissors. Isolated villi were mounted on coverslips and covered with light and solution penetrable polycarbonate membrane (GE, Minnetonka, MN). Coverslips were mounted on a perfusion chamber, placed on an inverted microscope, superfused with NH_4_^+^ buffer, followed by sequential perfusion with tetramethylammonium (130 mM TMA-Cl, 20 mM HEPES, 5 mM KCl, 1 mM TMA-PO_4_, 2 mM CaCl_2_, 1 mM MgSO_4_, and 25 mM glucose) and Na^+^ buffer that drives Na^+^-dependent pH recovery. Na^+^ buffer was supplemented with 30 μM HOE694 or 2 μM dimethyl amiloride to inhibit NHE1 and NHE2 activities. The microfluorometry was performed using the Metafluor software (Molecular Devices, Sunnyvale, CA) and two to three traces of Na^+^-dependent pH recovery were captured from each coverslip, each trace originating from an independent group of cells. For each experiment, a minimum of four coverslips per group were studied. Na^+^/H^+^ exchange rate was described by the initial rate of pH_i_ recovery, which was calculated by determining slopes along the pH_i_ recovery by linear least-squares analysis over a minimum of 7 s.

*Quantitative RT-PCR*. Total RNA was isolated from ileal mucosa scrapes using RNA Extraction Kit (Qiagen, Germantown, MD), and cDNA was synthesized using SuperScript III First-Strand Synthesis Kit (Life Technologies, Carlsbad, CA). Quantitative RT-PCR was performed as previously described using iQ SYGR Green Supermix (Bio-Rad, Hercules, CA) on the Eppendorf Mastercycler realplex (Eppendorf, Enfield, CT). The primers are as follows:

NHE1: 5′-TCT​TCA​CCG​TCT​TTG​TGC​AG-3′ and 5′-TAA​ACC​GGT​TGA​GCT​TGT​CC-3’. NHE2: 5′-CAG​TGT​CAG​CGA​AAC​CTT​GA-3′ and 5′-GGC​AAT​GAA​TGA​TGA​ACT​GGT​CCT-3’. NHE3; 5′-GAG​CTG​AAC​CTG​AAG​GAT​GC-3′ and 5′-CTC​CAG​AGA​CTG​CAT​GTC​CA-3’: *ß*-actin; 5′-AGC​CAT​GTA​CGT​AGC​CAT​CC-3′ and 5′-TCT​CAG​CTG​TGG​TGG​TGA​AG-3’.

### Co-Immunoprecipitation and Western Blot

Caco-2bbe/NHE3 cells were lysed in cold lysis buffer (Cell Signaling) containing 150 mM NaCl, 1 mM *ß*-glycerophosphate, 2.5 mM sodium pyrophosphate, 1 mM Na_2_EDTA, 1 mM EGTA, 1 mM Na_3_VO_4_, 1 μg/ml leupeptin, 1% Triton X-100, and protease inhibitors cocktail tablets (Roche, Indianapolis, IN). Lysate (500 μg) was pre-cleared by incubation with 30 µl of protein A-Sepharose beads for 1 h and the supernatant was then incubated overnight with anti-VSVG P5D4 antibodies. Immunocomplex was purified by incubating with 50 µl of protein A-Sepharose beads for 1 h, followed by three washes in lysis buffer and two washes in PBS. All the above steps were performed at 4 C or on ice. Beads were eluted with 2.5X Laemmli buffer. Cell lysates and eluted samples were separated by SDS-PAGE. Western immunoblotting was performed as previously described (No et al., 2014) and visualization were performed using the LICOR Odyssey Imaging System (Li-COR corp, Lincoln, NE). Densitometric analysis was performed using ImageJ software (National Institutes of Health).

### Detection of NHE3 Ubiquitination

Caco-2bbe/NHE3 cells were transiently transfected with pMT123 to express HA-Ub (Han and Yun, 2020). Cells were lysed in cold lysis buffer supplemented with 10 µM MG132 to inhibit proteasomal degradation. Equal amounts of cell lysates (typically 300 μg) were incubated overnight with anti-NHE3 EM450 antibody (Yoo et al., 2012), followed by incubation with protein A-Sepharose beads for 1 h. Immunocomplex was washed twice in lysis buffer and once in PBS. NHE3 was eluted, resolved by SDS-PAGE, and immunoblotted with anti-HA antibody.

### Surface Biotinylation

Surface biotinylation of NHE3 was performed as previously described (No et al., 2014). Briefly, cells were rinsed twice in PBS and incubated in borate buffer (154 mM NaCl, 7.2 mM KCl, 1.8 mM CaCl_2_, and 10 mM H_3_BO_3_, pH 9.0) for 10 min. Cells were then incubated for 40 min with 0.5 mg/ml sulfo-NHS-LC-biotin (Pierce, Rockford, IL) in borate buffer. Unbound sulfo-NHS-LC-biotin was quenched with Tris buffer (20 mM Tris, 120 mM NaCl, pH 7.4). Cells were lysed in lysis buffer (150 mM NaCl, 1 mM *ß*-glycerophosphate, 2.5 mM sodium pyrophosphate, 1 mM Na_2_EDTA, 1 mM EGTA, 1 mM Na_3_VO_4_, 1 μg/ml leupeptin, 1% Triton X-100, and protease inhibitors cocktail tablets) and sonicated for 2 x 15 s. Lysate was agitated for 30 min and spun at 14,000 x *g* for 30 min at 4°C to remove the insoluble cell debris. An aliquot was retained as the total fraction representing the total cellular NHE3. Protein concentration was determined and 1 mg of lysate was then incubated with streptavidin-agarose beads (Pierce) for 2 h. The streptavidin-agarose beads were washed 3 times in lysis buffer and twice in PBS. All the above procedures were performed at 4°C or on ice. Biotinylated surface proteins were then eluted by boiling the beads at 95°C for 10 min. Dilutions of the total and surface NHE3 were resolved by SDS-PAGE and immunoblotted with an anti-VSVG antibody as described above.

### Confocal Immunofluorescence Microscopy

Caco-2bbe/NHE3 cells grown on Transwells (Corning, Lowell, MA) were fixed using 100% methanol at −20°C for 20 min. Cells were washed three times with PBS, permeabilized using 0.05% Triton X-100 in PBS, and blocked with 5% normal goat serum for 1 h at RT. Cells were then stained with mouse anti-VSVG P5D4 antibody for 16 h at 4°C. Cells were washed with PBS three times and incubated with Alexa 488-conjugated goat anti-mouse IgG (Invitrogen) and Alexa Fluor 647 phalloidin (ThermoFischer) for 1 h at RT. After three 10 min washes with PBS, specimens were mounted with ProLong Glass Antifade Reagent (Invitrogen) and observed under a Nikon A1R HD confocal microscope (Nikon Instruments Inc., NY) coupled to a Plan Apo *λ* 60x Oli lens.

### Statistical Analysis

Statistical analysis was performed using independent samples two-tailed unpaired Student’s *t* test or ANOVA, followed by the Tukey post hoc analysis. Results are presented as mean ± SEM. Statistical analysis was performed using GraphPad Prism software (La Jolla, CA). A value of *p* < 0.05 was considered significant.

## Results

### Metformin Induces Diarrhea in Diabetic *db/db* Mice

**FIGURE 1:**
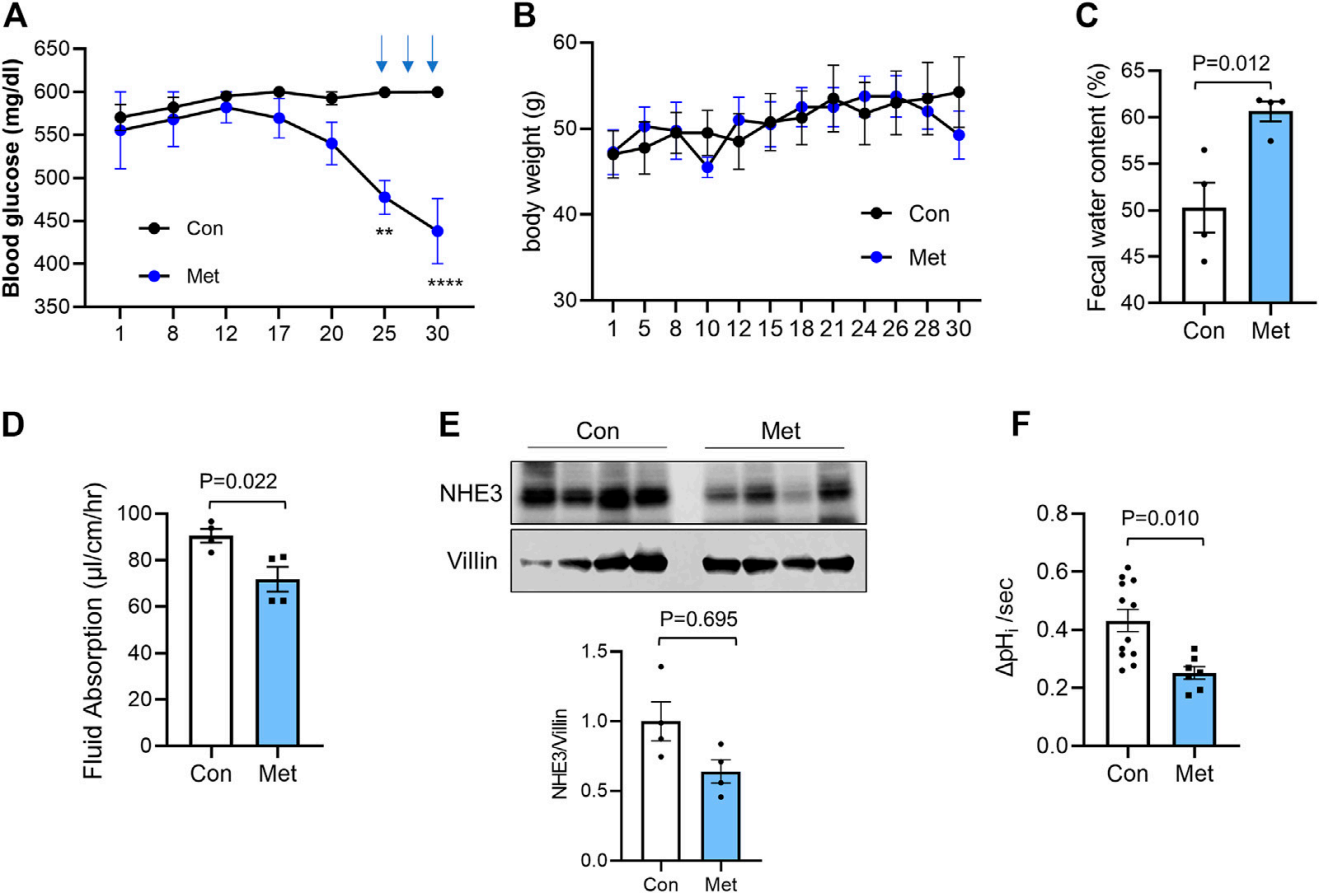
Metformin decreases intestinal water absorption in *db/db* mice Metformin was administered to *db/db* mice in drinking water (1 mg/ml) for 30 days. On day 25, 27, and 29, metformin (500 mg/kg) was administered once a day by gavage. Blood glucose **(A)** and body weights **(B)** were determined. Data are presented as mean ± SEM. *n* = 4. **, *p* < 0.01, ***, *p* < 0.001 by two-way ANOVA with Tukey’s post-hoc test. **(C)** Fecal water content was determined by weighing feces of control (Con) or metformin treated (Met) mice before and after drying. **(D)** Fluid absorption in the mouse ileum was determined by *in vivo* perfusion system in the presence of the CFTR inhibitor, CFTRinh-172. The rates of fluid absorption in control (Con) or metformin (Met) treated mice are shown. Data are presented as mean ± SEM. Statistical significance was determined by unpaired *t*-test. **(E)** Cellular expression of NHE3 in ileal mucosal lysates from *db/db* mice treated with metformin or not were determined. Villin was used as a loading control. Graph below shows NHE3 expression levels relative to villin. *n* = 4. **(F)** NHE3 activities were determined in isolated villi from control and metformin treated *db/db* mice. *n* ≥ 7 coverslips from two mice per group. Statistical significance was determined by unpaired *t*-test.

To assess whether metformin causes diarrhea, we first determined the effect of metformin in *db/db* T2D mice (Robinson et al., 2012). We started the treatment of mice by providing metformin at the concentration of 1.0 mg/ml in drinking water for 4 weeks. We observed a slight change in the blood glucose levels that did not reach statistical significance compared with the untreated cohort. Takemori et al. (Takemori et al., 2020) recently published their metformin treatment scheme consisting of a stepwise escalation of metformin dosage which lowered plasma glucose levels in mice. Therefore, we incorporated a part of their treatment scheme by administrating high dose metformin (500 mg/kg/day) for three alternative days during the last week of our treatment scheme. This regiment of metformin administration resulted in a significant reduction in glucose levels although still at >400 mg/dL (Figure 1A). There was no significant difference in body weight between control and metformin-treated groups (Figure 1B). Although none of the mice developed watery diarrhea, the feces of metformin-treated mice appeared softer. Quantification of the water content of feces revealed that there was a significant increase in fecal water contents compared with control mice (Figure 1C).

We have previously demonstrated the use of *in vivo* perfusion through a small loop of intestine to determine NHE3-mediated absorptive process (Lin et al., 2010; He et al., 2015; Jenkin et al., 2018). Hence, to determine whether NHE3-mediated luminal water loss in the intestine, a proxy for diarrhea, is altered by metformin, we determine the rate of water flux in *db/db* mouse intestine. In addition to NHE3 regulating Na^+^ absorption, Cl^−^ secretion by CFTR is a major determinant of intestinal water loss (Field, 2003). Previous studies have shown that metformin inhibits protein kinase A (PKA)-mediated stimulation of Cl^−^ secretion by CFTR in the intestine (Rogers et al., 2013), suggesting that metformin-induced intestinal water loss is not mediated by CFTR. However, the effect of metformin on the basal CFTR activity is not clear and therefore mice were administered with the CFTR inhibitor CFTRinh-172 intraperitoneally 1 h prior to the start of perfusion to block CFTR-mediated secretion. The similar treatment was shown to block about 90% of CFTR activity (Ma et al., 2002; Thiagarajah et al., 2004). CFTRinh-172 is also shown to inhibit CLC-2 chloride channels that are expressed in IECs (Cuppoletti et al., 2014). Consistent with the increased fecal water content, metformin significantly lowered the rate of fluid absorption to 71.8 ± 5.4 μl/cm/h compared with 90.6 ± 2.9 μl/cm/h for the control group (Figure 1D). To determine whether NHE3 expression is altered by the prolonged treatment with metformin, mouse ileal mucosal layers were collected and NHE3 expression levels were compared with the expression levels of villin, which was used as a loading control for the epithelium. There was a trend that NHE3 expression relative to villin is decreased in metformin-treated mice, but it did not reach statistical significance (Figure 1E). To verify that metformin regulates NHE3, we determined the Na^+^/H^+^ exchange activity of NHE3 in isolated ileal villi as the rate of initial Na^+^-dependent intracellular pH (pH_i_) recovery (Lin et al., 2010; He et al., 2015). All NHE3 activity measurements were conducted in the presence of 30 µM Hoe-694, which inhibits endogenous NHE1 and NHE2 activities. NHE3 activity was reduced by about 39% in metformin-treated mouse intestine (Figure 1F). These data indicated that metformin inhibits NHE3 and NHE3-dependent water absorption in the intestine, but the regulation of NHE3 is most likely at the post-translational level.

### Metformin Inhibits NHE3 Activity

**FIGURE 2:**
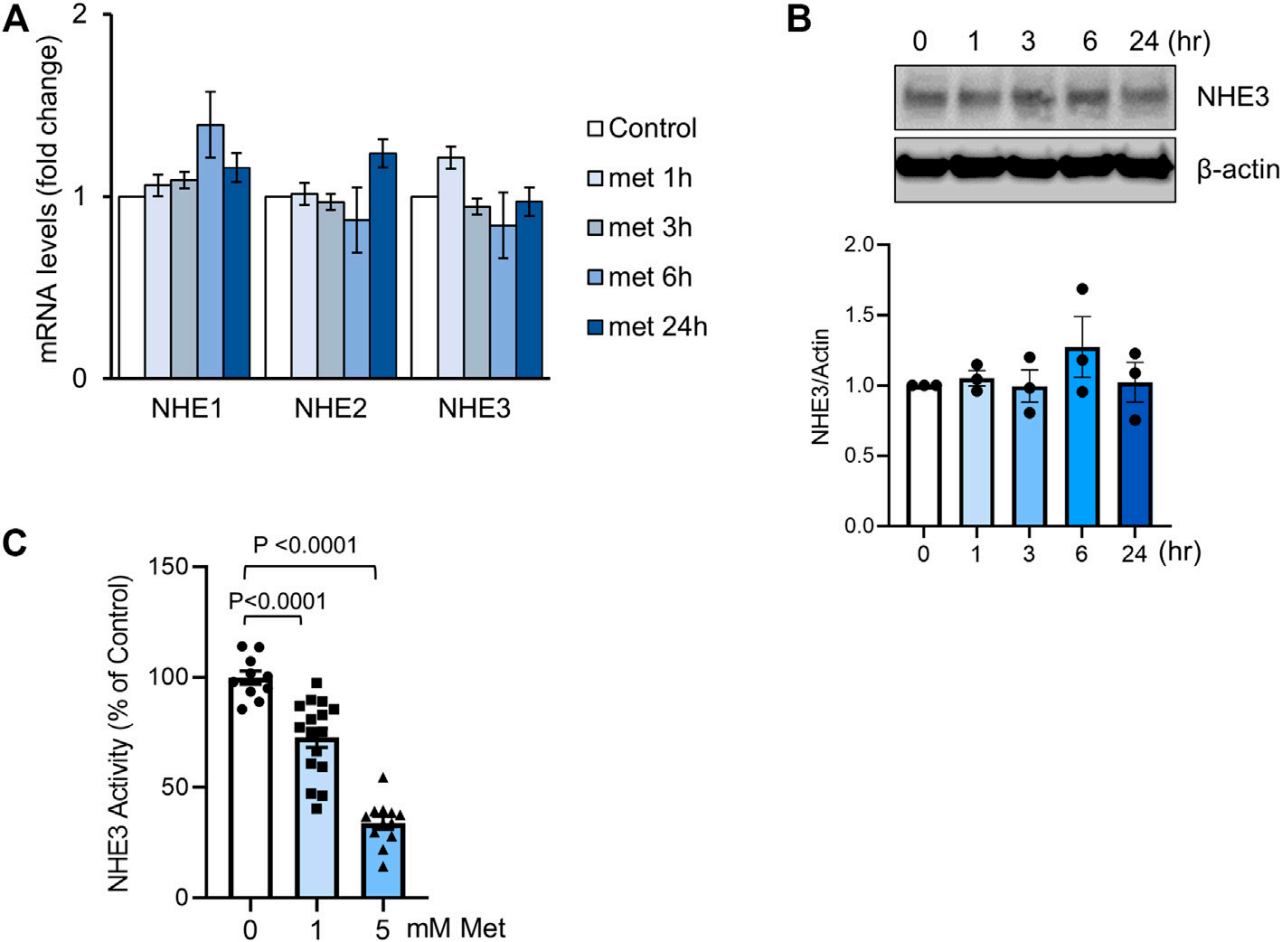
Metformin inhibits NHE3 activity. **(A)** SK-CO15 cells were treated with metformin for indicated time duration. Transcript levels (mean ± SEM) of NHE1, NHE2 and NHE3 were determined by RT-PCR. *n* = 9. **(B)** NHE3 protein expression in SK-CO15 cells treated with metformin was determined. *ß*-actin was used as a loading control. Graph below shows NHE3 expression levels relative to *ß*-actin. *n* = 3. **(C)** NHE3 activity was determined by measuring the initial rates of Na^+^-dependent changes in intracellular pH (pH_i_) in Caco-2bbe cells expressing human NHE3. Cells were treated with 1 or 5 mM metformin for 30 min. Results are presented as percent (mean ± SEM) of NHE3 activity at 0 mM metformin. *n* = 11–17. One-way ANOVA.

To confirm the lack of effect on NHE3 expression by metformin, we determined the effect of metformin on NHE3 transcript and protein expression in SK-CO15 cells, which express NHE3 endogenously (Yoo et al., 2012). Cells were treated with 1 mM metformin and NHE3 mRNA levels was determined by quantitative RT-PCR (Figure 2A). For up to 24 h, we did not observe a significant change in NHE3 mRNA levels. We also found that NHE1 and NHE2 transcript levels in SK-CO15 cells were not changed by metformin although the roles of NHE1 and NHE2 in diarrheal disease are unclear. Consistent with the findings in *db/db* mice, NHE3 protein expression was not significantly altered at 24 h of metformin (Figure 2B). We next determined whether the NHE3 activity is regulated by metformin. For this purpose, we utilized human colonic epithelial Caco-2bbe cells that are stably transfected to express human NHE3 with a VSVG epitope tag (Lin et al., 2010). The advantages of using Caco-2bbe cells are two-fold. First, Caco-2bbe cells lack endogenous NHE3 activity so that the regulation of ectopically expressed NHE3 is independent of transcriptional regulation. Second, the epitope-tagging of the exogenous NHE3 with a VSVG tag allows a better assessment of NHE3 protein by biochemical assays (Lin et al., 2010; Yoo et al., 2012). Na^+^/H^+^ exchange activity of NHE3 was determined in the presence of 30 µM Hoe-694 or 2 µM dimethyl amiloride to block endogenous NHE1 and NHE2 activities. Treating the cells with 1 mM metformin resulted in a significant decrease in NHE3 activity, which was augmented with 5 mM metformin (Figure 2C).

**FIGURE 3:**
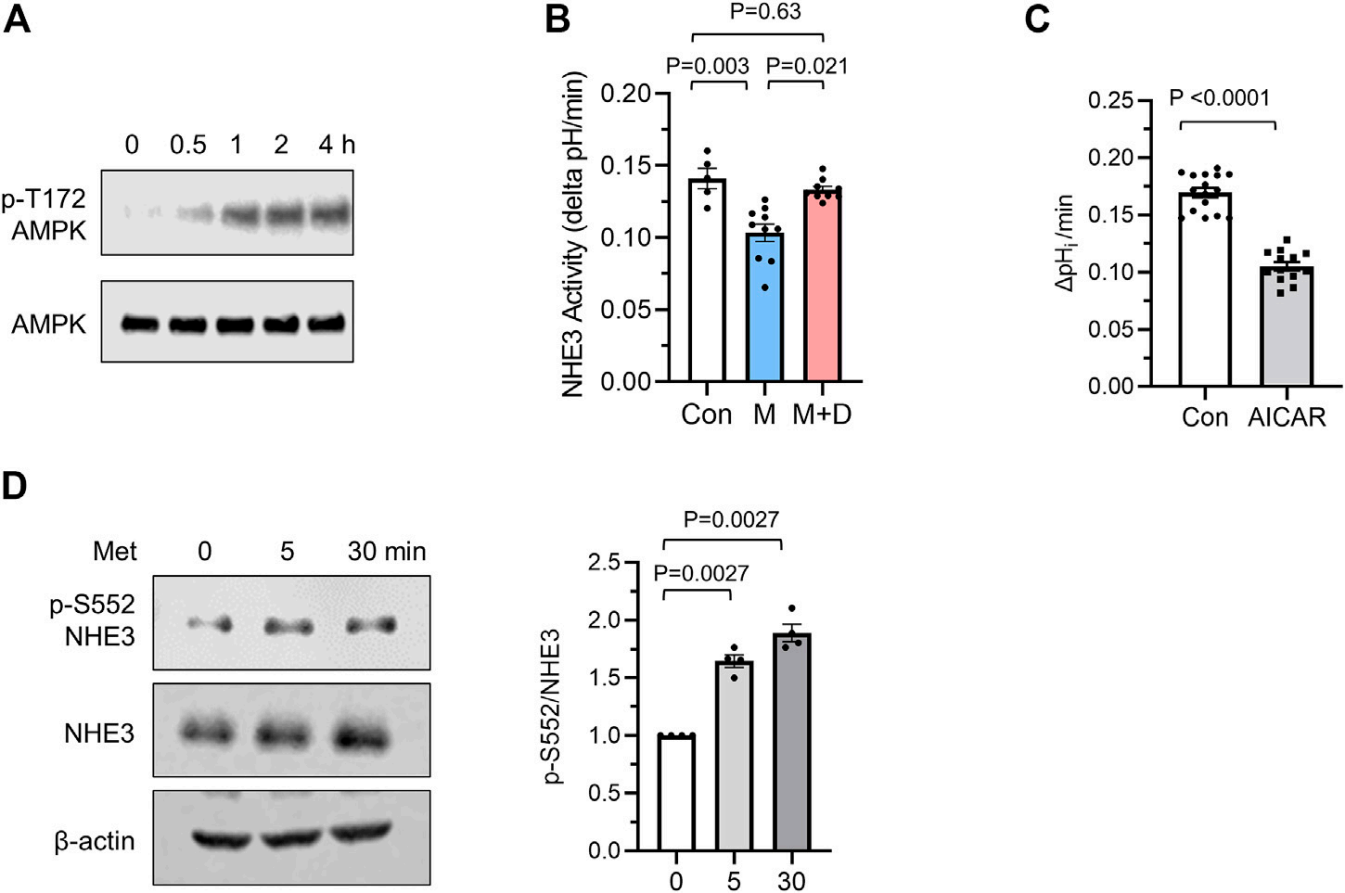
Metformin inhibits NHE3 activity. **(A)** Phosphorylation of AMPK at Thr172 was determined in Caco-2bbe cells treated with 1 mM metformin. **(B)** NHE3 activities were determined in untreated control (Con) cells, and cells treated with metformin (M) or metformin + dorsomorphin (M + D). *n* = 10. Statistical analysis was performed by two-way ANOVA with Tukey’s post-hoc test. **(C)** NHE3 activities in control and AICAR treated cells are shown. *n* = 11. Statistical significance was determined by unpaired *t*-test. **(D)** NHE3 phosphorylation at Ser552 was determined using phospho-specific antibodies. Cellular NHE3 and *ß*-actin were used as loading controls.

AMPK is the primary effector of metformin, but AMPK-independent action of metformin is also known (Foretz et al., 2010; Rena et al., 2017). We confirmed the activation of AMPK by metformin by determining phosphorylation of AMPK at Thr172 (Zhang et al., 2009), which serves as an indicator of AMPK activation (Figure 3A). To verify that NHE3 regulation by metformin is mediated via AMPK, cells were preincubated with the AMPK inhibitor dorsomophin, also known as Compound C, for 30 min prior to treatment with metformin. NHE3 inhibition by metformin was antagonized by dorsomorphin (Figure 3B), indicating that this regulation is mediated by AMPK. Consistent with these results, 5-aminoimidazole-4-carboxamide ribonucleoside (AICAR), which activates AMPK through accumulation of the AMP mimetic, AICAR monophosphate (Corton et al., 1995), induced inhibition of NHE3 (Figure 3C).

A number of studies have demonstrated that phosphorylation of rat NHE3 is important for inhibition of NHE3 activity (Kurashima et al., 1997; Zhao et al., 1999; Hu et al., 2001). Although the most previous studies have demonstrated phosphorylation of NHE3 at Ser552 and Ser605 by protein kinase A (PKA), recent studies have implied that NHE3 is a subject of phosphorylation at Ser552 by a mechanism independent of PKA (Liu and Jose, 2013; Chen et al., 2015). Therefore, we examined whether NHE3 is phosphorylated by metformin using phospho-specific NHE3 antibodies that recognize NHE3 only when phosphorylated at Ser552 (Kocinsky et al., 2005). We found that metformin increased NHE3 phosphorylation at Ser552 (Figure 3D). Because the anti-phospho-Ser605 antibody does not detect NHE3 in Caco-2bbe cells, we did not determine the status of phosphorylation at Ser605 (Jenkin et al., 2021).

### Metformin-Mediated Regulation of NHE3 is Nedd4-2 Dependent

**FIGURE 4:**
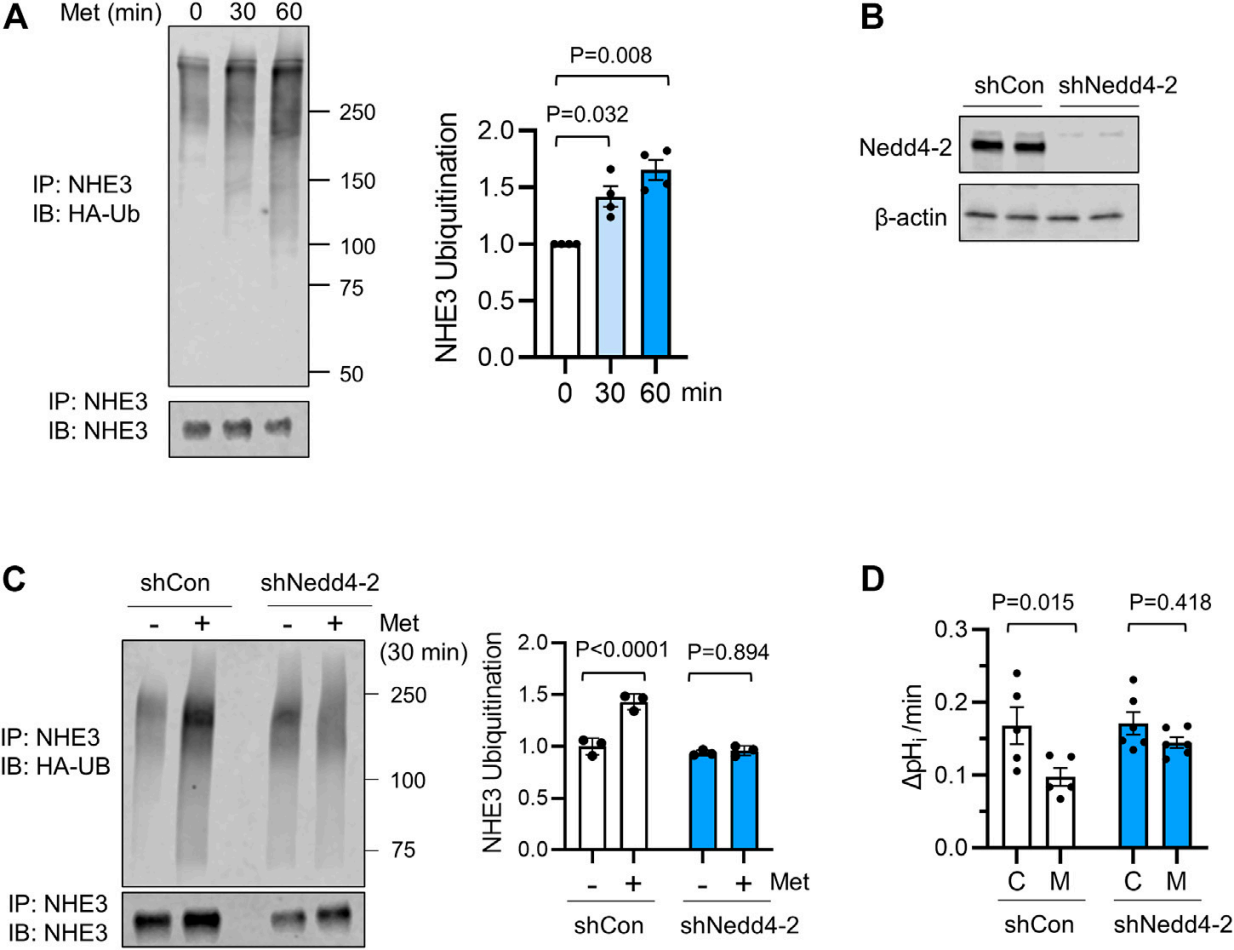
Ubiquitination of NHE3 by metformin is dependent on Nedd4-2. **(A)** Ubiquitination of hNHE3 was determined in Caco-2bbe cells expressing HA-Ub and treated with metformin. NHE3 was immunoprecipitated using anti-VSVG antibody and blotted with anti-Ub antibody. Lower panel shows immunoprecipitated NHE3. The graph on the right represents the quantification of Ub/NHE3 at 0, 30, and 60 min metfromin. *n* = 4. Statistical analysis by one-way ANOVA. The graph on the right represents the quantification of Ub/hNHE3. **(B)** Silencing of Nedd4-2 expression in Caco-2bbe/NHE3 cells transduced with shNedd4-2 is shown. **(C)** Ubiquitination of hNHE3 was determined as described earlier. The graph on the right represents the quantification of Ub/NHE3 in control (−) and metformin-treated (+) cells. *n* = 3. **(D)** NHE3 activity was determined as the rate of Na^+^-dependent pH_i_ recovery in cells expressing shCon or shNedd4-2 treated with metformin (M) or not (C). n ≥ 5. One-way ANOVA.

We have recently shown that NHE3s from certain species including human and non-human primates are a subject of ubiquitination (No et al., 2014; Jenkin et al., 2021). We therefore assessed NHE3 ubiquitination in Caco-2bbe cells. To determine ubiquitination levels, Caco-2bbe/NHE3 cells were transiently transfected to express HA-Ub. Following metformin treatment, NHE3 was immunoprecipitated and Ub levels of NHE3 was determined. As shown in Figure 4A, metformin treatment for 30 or 60 min resulted in a significant increase in NHE3 ubiquitination.

Nedd4-2 is a major Ub ligase regulating membrane proteins, including ENaC and NHE3 (Snyder et al., 2002; No et al., 2014). To determine whether NHE3 ubiquitination by metformin is dependent on Nedd4-2, Caco-2bbe cells were transduced with lentiviral shRNA specific for Nedd4-2, shNedd4-2 (Figure 4B) (No et al., 2014; Jenkin et al., 2021). Comparison of NHE3 ubiquitination induced by a 30 min treatment of metformin showed a marked decrease NHE3 ubiquitination levels with Nedd4-2 knockdown (Figure 4C). Importantly, silencing Nedd4-2 expression blocked metformin-induced inhibition of NHE3 (Figure 4D), demonstrating the functional relevance of Nedd4-2 in this regulation.

### Metformin Induces Retrieval of NHE3 From the Cell Surface

**FIGURE 5:**
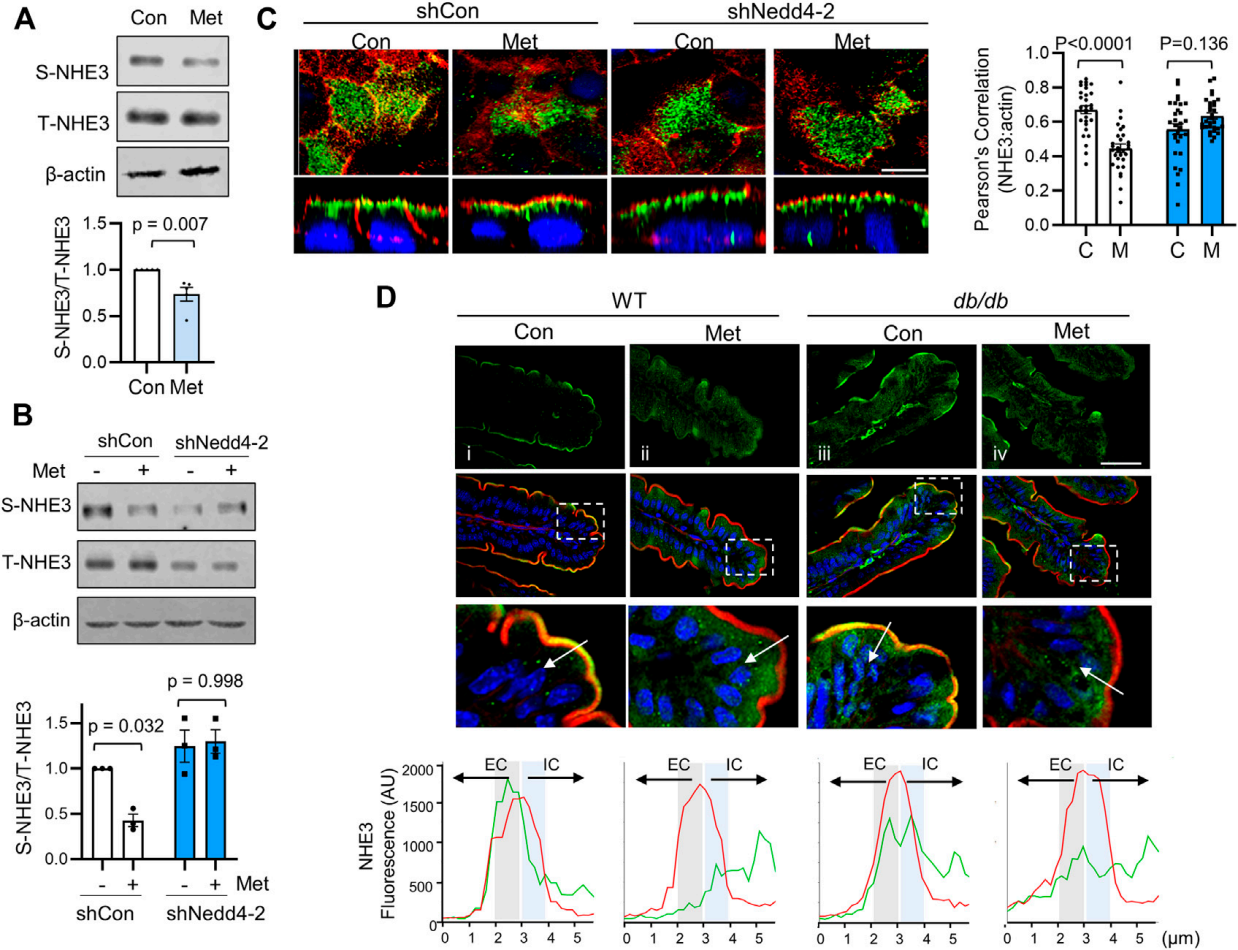
Metformin induces internalization of NHE3. **(A)** NHE3 expression at the apical membrane of Caco-2bbe cells treated with metformin was determined by surface biotinylation. The surface NHE3 expression was normalized to total NHE3 expression. The relative surface-to-total NHE3 ratios with the result in untreated cells set at 100% (mean ± SEM) are shown on the right. *n* = 5. **(B)** Apical membrane expression of NHE3 in cells transfected with shCon or shNedd4-2 was determined by surface biotinylation. **(C)** Caco-2bbe/NHE3 cells grown on Transwells were treated with metformin for 30 min. Top: horizontal (x–y) views at the cell surface. Bottom: cross-section (x–z) perpendicular to the confocal focal plane. Representative immunofluorescence labeling of NHE3 (green), actin (red, phalloidin), and nuclei (blue, DAPI) are shown. Two independent experiments with triplicates per group. Scale bar: 5 μm. Graph on the right represents Pearson’s correlation coefficient of NHE3 and *ß*-actin localization from 30 independent visual fields is shown. **(D)** Representative confocal IF images of ileal mucosa showing NHE3 (green), F-actin (red), and nuclei (blue) from WT and *db/db* treated with metformin for 3 days. For each condition, four mice, five sections/mouse examined. Scale bar: 5 μm. Bottom panels show magnified image of boxed area. Graphs below show representative fluorescent intensity of NHE3 (red) and F-actin (green) along a line drawn perpendicular to the membrane. Actin was used as a relative marker to standardize fluorescence intensity, with the peak of actin expression designated at the midway point across a 6 µm line. Extracellular (EC) and intracellular (IC) relative to F-actin.

Inhibition of NHE3 activity involves trafficking of NHE3 protein from the plasma membrane into an intracellular pool (Chow et al., 1999; Hu et al., 2001; Kocinsky et al., 2007; Hu et al., 2013; He et al., 2015). To determine whether metformin induces internalization of NHE3 protein, we determined NHE3 abundance at the apical membrane of Caco-2bbe cells by surface biotinylation before and after metformin treatment. As shown in Figure 5A, the abundance of NHE3 protein in the apical membrane was significantly decreased to 73.6 ± 7.4% of control levels without a change in the total amount of NHE3. In contrast, silencing of Nedd4-2 blocked the change in the apical NHE3 abundance (Figure 5B). This difference was corroborated by immunofluorescence (IF) confocal microscopy (Figure 5C) where Nedd4-2 knockdown mitigated endocytic trafficking of NHE3 by metformin.

Non-primate mammals, including mouse, express NHE3 that lacks a PY motif and hence NHE3 is not regulated via Nedd4-2-dependent ubiquitination (No et al., 2014). However, to determine whether the trafficking of NHE3 by acute treatment of metformin occurs *in vivo*, we treated WT and *db/db* mice by oral administration of metformin (500 mg/kg/day) for 3 days. In WT mice, NHE3 was primarily located at the terminal web of the microvilli region where IF signals of NHE3 and F-actin overlapped (Figure 5Di). Metformin resulted in a shift of NHE3 IF signals towards the intracellular region relative to F-actin (Figure 5Dii). In *db/db* mice, NHE3 IF signals were also detected at the brush border membrane. Unexpectedly, there was more prominent intracellular NHE3 signals in *db/db* mouse villi compared with WT villi under basal conditions (Figure 5Diii vs. i). Nevertheless, there was a shift of NHE3 fluorescent signals relative to actin by metformin in *db/db* mice (Figure 5Div), indicating that the endocytic trafficking of NHE3 from the brush border membrane occurs regardless of ubiquitination.

**FIGURE 6:**
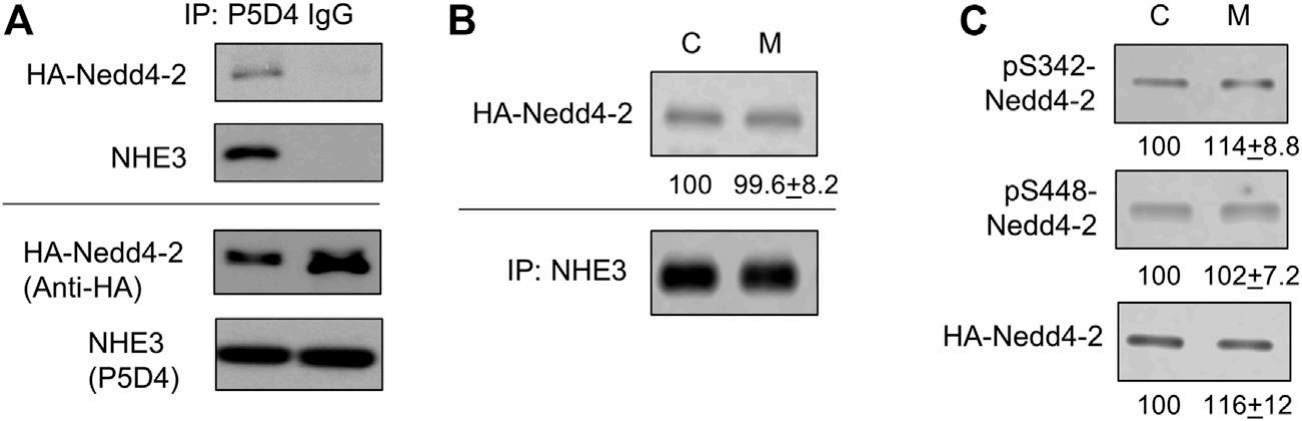
The interaction of Nedd4-2 with NHE3 is not enhanced by metformin. **(A)** Caco-2bbe cells expression NHE3 was lysed and immunoprecipitated with anti-VSVG P5D4 antibody or IgG. HA-Nedd4-2 co-immunoprecipitated in the presence of P5D4 but not IgG. **(B)** Co-immunoprecipitation of HA-Nedd4-2 and NHE3 are shown. NHE3 was immunoprecipitated from Caco-2bbe cells untreated (Con) and treated with 1 mM metformin (M) for 30 min. Co-immunoprecipitated Nedd4-2 was blotted with anti-HA antibody. Representative blots from three independent experiments are shown. Numbers below each blot represent quantification of protein bands relative to control. *n* = 3. **(C)** Phosphorylation of Nedd4-2 at S342 and S448 was determined using phosphorylation specific antibodies under the conditions descried above. Representative blots from three independent experiments are shown. Numbers below each blot represent quantification of protein bands relative to control. *n* = 3.

Given the Nedd4-2 dependent ubiquitination of NHE3, we next sought to determine whether AMPK activation promotes the interaction of Nedd4-2 with NHE3. To test this possibility, we expressed HA-Nedd4-2 in Caco-2bbe cells since anti-Nedd4-2 antibody is not suitable for immunoprecipitation based on our prior experience. The specificity of co-immunoprecipitation of HA-Nedd4-2 with NHE3 is demonstrated in Figure 6A where HA-Nedd4-2 co-immunoprecipitated with anti-NHE3 P5D4 antibody but not with IgG. To determine the effect of metformin on the Nedd4-2-NHE3 interaction, cells were treated with metformin for 30 min. Under basal conditions, HA-Nedd4-2 co-immunoprecipitated with NHE3(Figure 6B, *C*), but metformin did not result in a meaningful change in the amount of HA-Nedd4-2 co-immunoprecipitated with NHE3 (Figure 6B, *M*), indicating that metformin does not facilitate the interaction between Nedd4-2 and NHE3. Previous studies have demonstrated that a substrate interaction with Nedd4-2 is modulated via phosphorylation of Nedd4-2 at Ser342 or Ser448 (Debonneville et al., 2001; Ichimura et al., 2005; Zhou and Snyder, 2005). Therefore, we determined whether there is a metformin-dependent phosphorylation of Nedd4-2 at Ser342 and Ser448. We observed phosphorylation at Ser342 and Ser448 under basal conditions, but there was no change in phosphorylation levels in response to metformin (Figure 6C). These data indicate that the regulation of NHE3 by metformin is not dependent on a change in Nedd4-2 phosphorylation at Ser342 or Ser448.

## Discussion

Glycemic control is the key to the management of T2D and metformin has become the preferred blood glucose-lowering agent to manage T2D (Nyenwe et al., 2011; Rodriguez-Gutierrez and Montori, 2016; Bailey, 2017). However, the high incidence of adverse effects, including diarrhea, limits medication adherence amongst patients with T2D (Dandona et al., 1983; Foss and Clement, 2001; McGovern et al., 2018). The absorption of water results principally from the osmotic gradient created across the epithelium by absorption of electrolytes and nutrients. Reduced activity of NHE3, which mediates Na^+^ and water absorption in the intestine, causes diarrhea in human and rodents (Schultheis et al., 1998; Janecke et al., 2015; Xue et al., 2020). In this study, we found that metformin increased intestinal water loss, the hallmark of diarrhea, in a mouse model of T2D. Our study in Caco-2bbe cell demonstrated that metformin inhibited NHE3 activity with increased ubiquitination and reduced NHE3 abundance in the plasma membrane.

The *db/db* mouse model of leptin deficiency is currently the most widely used mouse model of T2D (King, 2012). However, the effects of metformin on blood glucose levels or diarrheal symptoms in *db/db* mice are not consistent. A previous study of administration of metformin (0.33 mg/ml) for 2 weeks in drinking water did not lower fasting blood glucose levels (Eskens et al., 2013), and we did not observe reduced glucose levels with 1.0 mg/ml metformin for 4 weeks. On the other hand, intragastric administration of metformin at 250 mg/kg/day for 2 weeks resulted in a significant drop (Han et al., 2017). Takemori and colleagues (Takemori et al., 2020) found that administering increasing amounts of metformin for 2 weeks to *db/db* mice not only lowered blood glucose levels but also resulted in diarrhea. Therefore, we incorporated intragastic delivery of metformin during the last week of metformin treatment based their study. We observed a significant drop in blood glucose without altering body weights. Although mice did not develop watery diarrhea, fecal water content was increased in metformin treated mice, indicating enhanced water loss in the intestine.

Recently, sex differences in adverse events associated with antidiabetic drugs have been reported (Joung et al., 2020). In this study, increased GI system disorder, which include diarrhea, nausea, and abdominal discomforts, was reported more frequently by women than men. However, this study did not determine whether diarrhea is specifically more often observed in women than in men. In another study, women experienced more frequent adverse effects associated with metformin than men, but no clear sex difference in diarrhea was observed (de Vries et al., 2020). Our studies of *db/db* mice used exclusively males and whether there is a sex difference in metformin-induced diarrhea requires further investigation.

More direct evidence for intestinal water loss was provided by the measurement of intestinal fluid absorption. Our data showed that metformin increased water loss in the intestine. We have shown previously that the water flux under the experimental conditions is mostly mediated by NHE3 and CFTR (Lin et al., 2010; Jenkin et al., 2021). Activation of CFTR by PKA is the major underlying cause of enterotoxigenic diarrhea (Field, 2003). However, a previous study has shown that AMPK inhibits Cl^−^ secretion by CFTR in mouse intestine (Rogers et al., 2013), thereby ruling out CFTR as a potential cause of metformin-induced diarrhea. Metformin increases the expression of SGLT1 in the rat intestine and increases glucose uptake by the enterocytes from the circulation (Bailey et al., 1994; Lenzen et al., 1996; Koffert et al., 2017). Others have shown that metformin inhibits transepithelial transport of glucose from the intestinal lumen into the blood (Horakova et al., 2019). The regulation of absorptive processes by SGLT1 and NHE3 are coordinated (Turner and Black, 2001; Lin et al., 2011), raising the possibility of indirect regulation of NHE3 via glucose transport by SGLT1. However, the perfusion media lacks glucose or amino acid so that any contribution by nutrient-coupled water absorption from the lumen is eliminated. Consistently, the Na^+^/H^+^ antiporter activity of NHE3 in isolated ileal villi was significantly reduced in metformin-treated mice further demonstrating that the effect on NHE3 is independent of SGLT1. Another potential target of metformin is ENaC, which mediates electrogenic Na^+^ absorption in the distal colon (Kunzelmann and Mall, 2002). Inhibition of ENaC by AMPK in renal and airway epithelial cells has been demonstrated (Carattino et al., 2005; Woollhead et al., 2005). However, ENaC is not present in the small intestine so that the increased water flux observed in our experiments is not mediated by ENaC. It remains to be determined whether AMPK inhibits colonic ENaC and whether ENaC contributes to metformin-induced diarrhea.

The pathophysiology of diarrhea is complex and multiple pathogenic mechanisms have been implicated for diabetic diarrhea, including autonomic neuropathy, bacterial overgrowth, and pancreatic exocrine insufficiency (Ogbonnaya and Arem, 1990). Other factors, such as bile acids and glucagon-like peptide 1 (GLP-1), are also known to contribute to metformin-mediated the adverse effects in the GI tract (McCreight et al., 2016). Considering that bile acids and GLP-1 are known to inhibit sodium and water absorption, these might amplify the contribution of NHE3 to diabetic diarrhea (Mekhjian and Phillips, 1970; Gordon et al., 1979; Carraro-Lacroix et al., 2009).

Regulation of NHE3 by protein kinases involves phosphorylation of NHE3 proteins. The inhibition of NHE3 by PKA is dependent on phosphorylation of NHE3 at Ser552 and/or Ser605 (Kurashima et al., 1997; Zhao et al., 1999), and cyclic GMP kinase II phosphorylates NHE3 at the same serines (Chen et al., 2015). Activation of NHE3 by SGK1 is associated with phosphorylation of NHE3 at S663 (Wang et al., 2005). In the current study, we found that metformin increased phosphorylation of NHE3 at Ser552. However, how the activation of AMPK by metformin might lead to phosphorylation of NHE3 is not clear. It seems unlikely that NHE3 phosphorylation by AMPK is mediated by PKA since AMPK works downstream of PKA (Medina et al., 2014). Another plausible mechanism involves protein kinase C (PKC). The activation of atypical protein kinase C (PKC) by AMPK has been previously demonstrated (Chen et al., 2002; Sajan et al., 2010), and a recent study of human renal proximal tubule cells demonstrated PKC-dependent phosphorylation of NHE3 (Liu and Jose, 2013). However, our recent findings suggest that phosphorylation at Ser552 is not necessary for the regulation of human NHE3 by PKA (Jenkin et al., 2021), and the functional importance of NHE3 phosphorylation at Ser552 by metformin in humans remains unclear.

We assumed that the effect of metformin, AICAR, and dorsomorphin is primarily mediated via AMPK based on the broad usage of these chemicals as a specific regulator of AMPK. However, AMPK-independent effects of metformin are known and other protein kinases, including ERK8, PHK, MELK, and Src, and LcK, can be inhibited by dorsomorphin (Bain et al., 2007; Foretz et al., 2010). As metformin, AICAR and dorsomorphin could all have off-target effects on other cellular pathways so that undisputable evidence that metformin regulates NHE3 via AMPK requires studies using IECs isolated from AMPKα/β-deficient animals.

The potential of substrate proteins with Nedd4-2 is often predicted by the presence of PPxY motifs although many other transporters and channels lacking PPxY are also shown to be regulated by Nedd4-2 (Manning and Kumar, 2018). The regulation of NHE3 by Nedd4-2 is species dependent in that only NHE3s of human and non-human primates possess PPxY motif, suggesting the evolutionary diversity in regulation of NHE3 (No et al., 2014). In the current study, we found that metformin increased ubiquitination levels of NHE3 in Caco-2bbe cells. Importantly, Nedd4-2 knockdown alleviated metformin-induced regulation of NHE3, demonstrating the critical role of Nedd4-2 in this regulation. Ubiquitination-dependent regulation of NHE3 is unique to human and non-human primates so that the regulation of NHE3 in *db/db* mice or other rodent models does not involves the ubiquitination process. Our recent study has demonstrated that the extent of intestinal water loss by cholera toxin and EPEC are magnified in humanized mice expressing human NHE3 in the intestinal tract (Jenkin et al., 2021). Therefore, we speculate that the effects of metformin on NHE3 and NHE3-dependent water absorption is greater in humans than in rodents.

Previous studies of the regulation of ENaC by PKA and SGK1 have shown that Nedd4-2 is a subject of phosphorylation at Ser342 and Ser448, which modulates the interaction of Nedd4-2 with ENaC (Debonneville et al., 2001; Snyder et al., 2004). *In vitro* phosphorylation and ^32^P-orthophophate labeling in HEK293 cells demonstrated that AMPK phosphorylates Nedd4-2 (Bhalla et al., 2006), prompting an expectation that metformin stimulates Nedd4-2 phosphorylation. More recently, direct phosphorylation of *Xenopus* Nedd4-2 at Ser444 (equivalent to Ser448 in human Need4-2) by purified AMPK *in vitro* has been demonstrated (Ho et al., 2018). However, we detected neither a change on Nedd4-2 phosphorylation nor enhanced interaction between Nedd4-2 and NHE3 by metformin. We showed recently that activation of PKA by forskolin increased Nedd4-2 phosphorylation at Ser342. However, the attempt to determine Nedd4-2 co-immunoprecipitated with NHE3 using antibody against Nedd4-2 did not show enhanced interaction between these two proteins. On the other hand, co-immunoprecipitation of pSer342-Nedd4-2 with NHE3 was specifically augmented by forskolin (Jenkin et al., 2021). An analysis of Nedd4-2 phosphorylation by mass spectrometry revealed additional phosphorylation sites to which anti-phospho-specific antibodies are not available (Hallows et al., 2010). Hence, it is conceivable that AMPK phosphorylates Nedd4-2 at sites other than Ser342 or Ser448. Alternatively, given AMPK-independent effects of metformin, other kinases regulated by metformin might mitigate Nedd4-2 phosphorylation complicating the interpretating of the results. Nevertheless, how metformin stimulates Nedd4-2-dependent ubiquitination of NHE3 remains unresolved. The Nedd4 family of HECT E3 ligases, including Nedd4-2, are composed of a C2 domain, three to four WW domains that interact with the PY motifs of target proteins, and a C-terminal ubiquitin ligase HECT domain (Rotin and Kumar, 2009). X-ray crystal structures of various NEDD4 family HECT domains have revealed that they exist in two distinct conformations that are believed to important for the catalytic activity of the enzyme turnover (Verdecia et al., 2003; Maspero et al., 2011). Others have shown that the HECT domain modulates self-ubiquitination of Nedd4-2 (Bruce et al., 2008). Recent studies showed that tyrosine phosphorylation of the C2 and HECT domain promotes the Ub ligase activity by unlocking the auto-inhibited state (Persaud et al., 2014; Grimsey et al., 2018). Therefore, we speculate that AMPK indirectly phosphorylates Nedd4-2 that alters the protein conformation to promote its catalytic activity towards NHE3.

In summary, we have demonstrated that metformin increases intestinal water loss through inhibition of NHE3-dependent fluid absorption. The inhibition of NHE3 by metformin is mediated by Nedd4-2-dependent ubiquitination of NHE3. Ubiquitination facilitates retrieval of NHE3 from the brush border membrane, resulting in decreased Na^+^/H^+^ exchange activity. NHE3 ubiquitination is species dependent and this mechanism of NHE3 pertains to humans and not rodents. A future study is needed to explore the regulatory mechanism of non-human by metformin.

## Data Availability

The original contributions presented in the study are included in the article/Supplementary Material, further inquiries can be directed to the corresponding author.

## Abbreviations

AICAR, 5-aminoimidazole-4-carboxamide ribonucleoside; AMPK, 5’-AMP-activated protein kinase; CFTR, cystic fibrosis transmembrane regulator; ENaC, epithelial sodium channel; HA, hemagglutin; IEC, intestinal epithelial cell; Nedd4-2, neural precursor cell expressed, developmentally down-regulated 4-2; NHE3, Na+/H+ exchanger 3; pHi, intracellular pH; PKA, protein kinase A; SGLT1, sodium-coupled glucose cotransporter 1; shRNA, short hairpin RNA; T1D, type 1 diabetes; T2D, type 2 diabetes; Ub, ubiquitin; VSVG, vesicular stomatitis virus glycoprotein.
